# Analysis of end-of-life treatment and physician perceptions at a university hospital in Germany

**DOI:** 10.1007/s00432-021-03652-0

**Published:** 2021-05-05

**Authors:** Nicole Heerde, Wolf-Karsten Hofmann, Ralf-Dieter Hofheinz, Sylvia Büttner, Deniz Gencer

**Affiliations:** 1 Third Department of Medicine, Medical Faculty Mannheim, University Medical Center Mannheim, Heidelberg University, Theodor-Kutzer-Ufer 1-3, 68167 Mannheim, Germany; 2 Department of Medical Statistics, Biomathematics and Information Processing, University Medical Center Mannheim, Heidelberg University, Heidelberg, Germany

**Keywords:** Palliative treatment, End of life care, Dying patients, Pain management

## Abstract

**Purpose:**

Providing state-of-the-art palliative care is crucial in all areas of in- and outpatient settings. Studies on the implementation of palliative care standards for dying patients are rare.

**Methods:**

*N* = 141 physicians from all internal departments were polled anonymously about the treatment of dying patients using a self-designed questionnaire. Furthermore, we evaluated the terminal care of *n* = 278 patients who died in internal medicine departments at University Hospital Mannheim between January and June, 2019 based on clinical data of the last 48 h of life. We defined mandatory criteria for good palliative practice both regarding treatment according to patients' records and answers in physicians' survey.

**Results:**

Fifty-six physicians (40%) reported uncertainties in the treatment of dying patients (*p* < 0.05). Physicians caring for dying patients regularly stated to use sedatives more frequently and to administer less infusions (*p* < 0.05, respectively). In multivariate analysis, medical specialization was identified as an independent factor for good palliative practice (*p* < 0.05). Physicians working with cancer patients regularly were seven times more likely to use good palliative practice (*p* < 0.05) than physicians who did not. Cancer patients received good palliative practice more often than patients dying from non-malignant diseases (*p* < 0.05).

**Conclusion:**

Guideline-based palliative care for dying patients was found to be implemented more likely and consistent within the oncology department. These results point to a potential lack of training of fellows in non-oncological departments in terms of good end-of-life care.

**Supplementary Information:**

The online version contains supplementary material available at 10.1007/s00432-021-03652-0.

## Introduction

During the last decades, palliative medicine has been implemented as an independent medical specialty (Buss et al. 2017). However, the palliative and terminal care of patients (pts) with malignant and non-malignant diseases are crucial in all areas of inpatient and outpatient care, leading to improved quality of life as for instance shown for patients with chronic heart failure (Rogers et al. 2017). Several studies indicate that palliative care (PC) should be integrated into standard practice earlier and more consistently, not only in cancer patients (Beaussant et al. 2018; Breuer et al. 2011; Verhofstede et al. 2017; Hua and Wunsch 2014; Gelfman et al. 2017; Holden et al. 2020). Studies on the implementation of PC standards for dying pts are rare, for Germany basically no data have been published thus far. The German S3 Guideline for Palliative Medicine provides recommendations on treating dying pts suffering from incurable cancer (Leitlinienprogramm Onkologie 2019).

In 2014 Clemans and coworkers reported that terminally ill pts with non-malignant diseases were less often treated with the goal of palliation (2014).

Studies point to barriers like fear of drug addiction or ceiling effects of medication as well as opioid side effects both in physicians and pts. This could lead to suboptimal symptoms or pain control (Makhlouf et al. 2019; Smallwood et al. 2017; Hadjiphilippou et al. 2014). It was hypothesized that medical specialty and experience seemed to play a major role in this regard (Kim et al. 2011).

The current study sought to examine physicians’ attitudes and knowledge about PC and to evaluate the actual treatment offered to dying pts in different departments of internal medicine at the University Hospital Mannheim.

## Methods

### Survey of physicians

Physicians from all departments for internal medicine at the University Hospital Mannheim were asked to fill in a self-designed questionnaire confidentially between August to October 2019. The physicians were personally approached and reminded by the authors to return the questionnaire. The anonymously answered questionnaires were collected in a collection box (to ensure anonymity). Eleven physicians on parental leave or working in other facilities at the time of the survey period were excluded.

Due to the lack of standardized questionnaires on our topic in literature, we developed one by ourselves. In the first part, statistical information was requested such as gender, position (fellows, attendings or senior physicians), department and frequency of care for dying pts. We differentiated between 4 departments: cardiology, gastroenterology/geriatrics, oncology and nephrology. Since gastroenterology and geriatrics were merged into one department at the time of our study, a separate analysis was not possible. Furthermore, in our analysis of physicians’ questionnaires we could not clearly differentiate if the physicians were working on the palliative care unit and the intensive care unit because we only asked about the department they were working at and not about the specific medical ward. As a matter of fact, the palliative care unit belongs to the oncological, the intensive care unit to the cardiological department.

The second part of the questionnaire was designed to evaluate knowledge about palliative medicine in medical school and further education. For the most part, we asked for information about knowledge, attitudes, competence, and experience with dying pts. For detailed information about the questionnaire see supplement 1.

### Analysis of patient records

Furthermore, data about treatment during the last 48 h before the death of pts between January and June 2019 in the departments of internal medicine at the University Hospital Mannheim (*n* = 374) were evaluated retrospectively on the basis of pts records. All patients were included in our analysis who were documented to be in the process of dying within the last 48 h of life (*n* = 278). In the following, these pts are named as dying pts. Pts with a sudden or unexpected death (*n* = 3) and those who died after resuscitation (*n* = 85) were excluded from the analysis. No recording was available in 8 pts. Out of 136 pts who died in the intensive care unit (ICU) in the examined period at all, 66 pts met the inclusion criteria. For these 66 pts it was specified by the therapeutic team not to resuscitate them and it was documented that these pts should receive just the best supportive care.

The pts’ records were available digitally archived on the hospital server. The authors analysed the pts records regarding administered medication (in the curve sheet), procedures, diagnostic measures and symptoms documented by physicians and nurses in the physicians’ visit or nursing documentation.

**Fig. 1:**
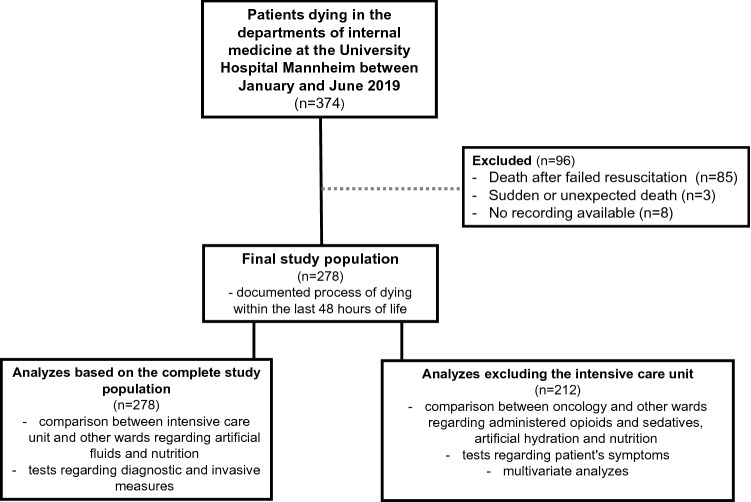
Selection of patients and patient population included in statistical analyses

Due to different treatment standards in terms of analgo-sedation and other needs (e.g., because of continued intubation) a comparison between ICU with non-ICUs regarding administered opioids and sedatives was considered not reasonable. The ICUs also were excluded from multivariate analyzes and from analysis of documented symptoms due to a different documentation system. The entire study population is considered in comparison between the intensive care unit and other wards regarding artificial fluids and nutrition and in tests regarding diagnostic and invasive measures. A flow chart of the patient selection and patient population included in the different statistical analyzes can be found in Fig. 1.

When analyzing the pts’ records we were able to differentiate between all medical departments and it was also possible to analyze the data of the pts treated on the palliative care unit or the intensive care unit separately.

### Statistical evaluation

The data were analysed by standard statistical tests including Chi-Square test, Fisher’s exact test, Student's *t*-test, Cochran–Armitage Trend Test. For logistic regression analysis we defined parameters which are meant to be mandatory to fulfil criteria for good palliative practice regarding the physician’s survey (GPP) as well as for the patient records (GPP*). Based on the symptom prevalence during the dying phase found in literature, including pain, restlessness, dyspnea and anxiety (Albert 2017; Ellershaw et al. 2001), we defined parameters to be fulfilled regarding knowledge and attitude of physicians when treating dying pts providing GPP: knowing the WHO guidelines for the pain to ensure adequate pain control, less fear of opioid side effects to minimize reservations against opioids in general, using sedatives to control other symptoms that could occur in dying patients e.g. agitation/anxiety, to reduce or discontinue parenteral therapies like it is demonstrated to be beneficial in literature (Morita et al. 2005; Bruera et al. 2005) and recommended in guidelines for palliative care (Leitlinienprogramm Onkologie 2019; Cheshire and Merseyside Palliative and End of Life Care Network Audit Group 2017). GPP* was fulfilled if both, pts were treated with opioids—which is recommended to treat symptoms such as pain but also explicitly dyspnea and anxiety in dying pts (Leitlinienprogramm Onkologie 2019)—and received <1.5 L parenteral hydration (Morita et al. 2005; Bruera et al 2005).

A test result was considered statistically significant if *p* ≤ 0.05. All analyses were performed using the SAS statistical analysis software (release 9.4; Cary, NC, USA).

## Results

### Survey on knowledge and attitudes of physicians

#### Evaluable questionnaires and physicians’ characteristics

A total of *n* = 141 questionnaires were evaluable, which corresponds to a response rate of 97%. Physicians’ characteristics are depicted in Table 1.

**Table 1:** Characteristics of *n* = 141 physicians polled at the departments for internal medicine (cumulative numbers may differ due to missing data entries)

	*n*	%
All physicians	141	100
Gender		
Male	83	59
Female	58	41
Working in medical department		
Cardiology	56	40
Gastroenterology, geriatrics	35	25
Oncology (including palliative care unit)	28	20
Nephrology	21	15
Position		
Fellow	77	55
Attending	19	13
Senior physician	45	32
Frequency of treating dying patients		
Daily	9	6.4
At least once a week	45	32
At least once a month	56	40
Less than once a month	30	21

#### Palliative care in education, attitudes and knowledge about the care of dying pts

Fellows rated their PC education less often inadequate (69%, *n* = 53) than attendings (84%, *n* = 16) or senior physicians (96%, *n* = 43) (*p* < 0.01). *N* = 56 expressed frequent uncertainties when treating dying pts, 73% of them (*n* = 41) were fellows (*p* < 0.01).

The majority of physicians stated to use opioids for the treatment of pain (96%, *n* = 136) as well as for management of dyspnoea or anxiety (94%, *n* = 132) in dying pts. 73% (*n* = 102) also stated to use other medication for palliative sedation.

**Fig. 2:**
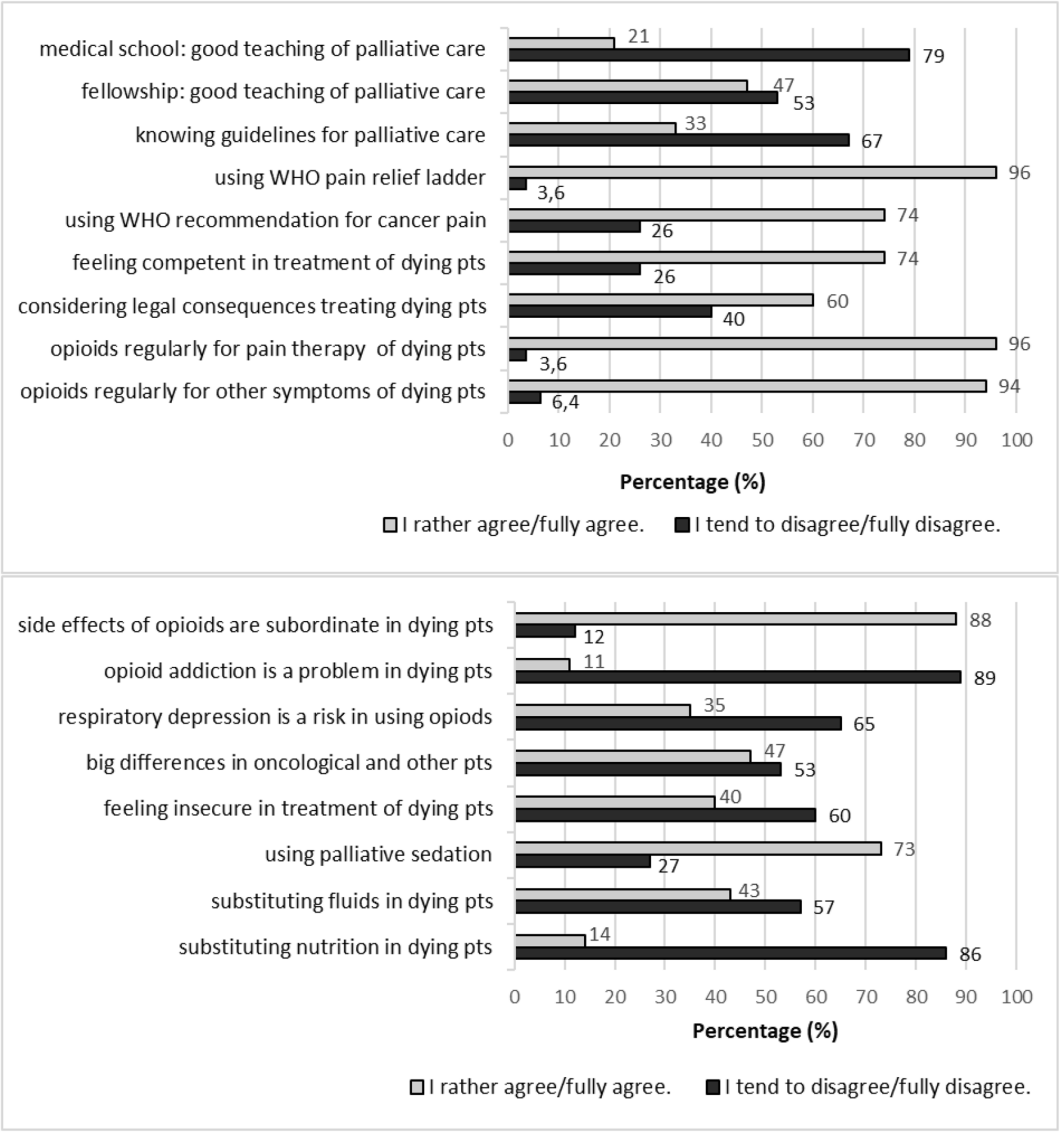
Physicians (*n* = 141) polled at the departments for internal medicine—answers in the questionnaire on dealing with dying patients

Most physicians considered opioid side effects (88%, *n* = 124) and the risk of addiction (89%, *n* = 126) to be less important in the treatment of dying pts. However, about one-third (35%, *n* = 49) stated to use opioids very carefully due to the risk of respiratory depression. Furthermore, almost half of the physicians (47%, *n* = 64) indicated to make different end-of-life treatment decisions based on the presence or absence of malignant disease in pts. 43% (*n* = 59) responded to continue artificial hydration and 14% (*n* = 20) to apply artificial nutrition in the dying phase (see Fig. 2).

#### Further univariate and multivariate analyses

Significant differences in terms of physician’s knowledge and perceptions were noticed regarding the affiliation to the four medical departments. Oncologists rated their further training in PC to be better (*p* < 0.01) and were more familiar with the German S3 Guideline for Palliative Medicine (*p* = 0.01). They felt more confident and competent in treating dying pts (*p* = 0.02), feared less legal consequences in terms of treatment limitation (*p* < 0.01) and were less afraid of opioid addiction of the pts (*p* < 0.01). They also more often stated to use sedative medications (*p* = 0.02) and disagreed to parenteral administration of fluids (*p* < 0.01) and nutrition in dying pts (*p* = 0.04).

Approximately half of the fellows (53%, *n* = 41) expressed frequent uncertainty in the treatment of dying pts. This applied only to 16% (*n* = 3) of the attendings and 27% (*n* = 12) of the senior physicians (*p* < 0.01). In particular, sedatives were used less often by fellows (*p* < 0.01). Physicians caring for dying pts on a regular basis, tended to use sedatives more frequently for symptom management (*p* = 0.01) and to administer less infusions (*p* = 0.01).

Logistic regression revealed the medical department to be an independent factor for GPP (*p* = 0.01). Physicians working in oncology were seven times more likely to use GPP than those working in the cardiology (95% CI 2.3–22.3; *p* < 0.01). Results of the logistic regression are depicted in Table 2.

**Table 2:** Multivariate analysis—“Good palliative practise^a^ among *n* = 141 physicians polled at the departments for internal medicine”

Parameter	Estimate	Standard error	Wald Chi^2^	Pr > Chi^2^
Medical department: oncology	1.9458	0.5846	11.0761	0.0009

^a^At least one of the following criteria had to be fulfilled for good palliative practice: knowing the WHO opioid guidelines, less fear of opioid side effects, use of sedatives for symptom management, diminution of parenteral therapies in the dying phase

### Analysis of patient records

#### Pts’ characteristics

The mean age of the *n* = 278 dying pts was 75.4 years (median 79 years, range 23–101). The pts’ characteristics are shown in Table 3.

**Table 3:** Characteristics of *n* = 278 patients who died expectedly in the departments of internal medicine

	*n*	%
All patients	278	100
Treated in		
Cardiology (periphere ward)	27	9.7
Intensive care unit	66	24
Gastroenterology	39	14
Geriatrics	26	9.4
Palliative care (PC) unit	68	24
Oncology (except PC unit)	35	13
Nephrology	17	6.1
Primary disease		
Malignant disease	124	45
Non-malignant disease	154	55

#### Medication and treatment

For an overview of administered medication see supplement 2. 78% of all pts were treated with opioids (*n* = 217), 34% received (*n* = 95) sedatives. The majority of pts (80%, *n* = 53) treated at the ICU received continuous opioid therapy.

The Intensive care unit is excluded in the following analyses in this section. 90% (*n* = 93) of pts in the oncology department received opioids at the end of life, while 61% (*n* = 66) of pts in other departments did so (*p* < 0.01). 43% (*n* = 44) of pts in oncology were treated with sedatives compared to 14% (*n* = 15) in other departments (*p* < 0.01). Only 13% (*n* = 15) of pts dying of malignant diseases were not treated with opioids while 39% of the pts with non-malignant diseases did not receive opioids (*n* = 38; *p* < 0.01). A high percentage of pts treated with sedatives (75%, *n* = 44) suffered from malignant diseases (p < 0.01). The majority of pts receiving butyl-scopolamine to relieve death rattle were treated in oncology (93%, *n* = 28) (*p* < 0.01). The proportion of pts who received opioids (17%, *n* = 11; *p* < 0.01) and sedatives (26%, *n* = 9; *p* = 0.03) solely in the last 24 h of life was lower at PC unit in comparison to all other wards. Pre-existing oral medication was discontinued more frequently and continuous intravenous administration of opioids was more common in oncology (*p* < 0.01, respectively). Altogether, *n* = 47 pts were treated with continuously intravenously or subcutaneously administered morphine with an average dose of 1.4 mg/h (median 0.9 mg/h) in oncology. Pts treated with continuously applied morphine in all other departments (*n* = 28) received a higher average dose (2.4 mg/h; median 2 mg/h; *p* < 0.01).

#### Artificial hydration and nutrition

Majority of pts (*n* = 216, 78%) received artificial hydration, while artificial nutrition was used in about a third of pts (*n* = 94, 34%). Pts treated in PC unit received both less artificial fluids (*p* = 0.02) and less nutrition (*p* < 0.01) than in all other peripheral wards.

While 91% (*n* = 60) of the pts treated in an ICU received artificial hydration and 58% (*n* = 38) artificial nutrition, in other wards this related only to 74% (*n* = 156, *p* < 0.01) and 26% (*n* = 56, *p* < 0.01).

#### Diagnostic and invasive measures

A summary of the diagnostic and invasive measures is given in supplement 3. Differences between the medical specialties were found as follows: In oncology laboratory tests were performed in 48% (*n* = 49), in the other departments in 86% (*n* = 150) of the pts (*p* < 0.01). In oncology, 15% (*n* = 15) received imaging, in the other departments about half of the pts (51%, *n* = 89; *p* < 0.01). With regard to ultrasound there could be found similar results (*n* = 77; in oncology 5.8%, *n* = 6; in the remaining departments 41%, *n* = 71; *p* < 0.01). Substitution of blood components was carried out for the minority of pts at the end of life (18%, *n* = 51) and only 5.0% (*n* = 14) had surgery, most of them at ICU. Pts who did not receive any of these measures were treated significantly more often in the PC unit (*p* < 0.01).

#### Patient's symptoms as documented in medical records

Symptoms of dying pts documented by physicians and nurses are illustrated in supplement 4. Most common symptoms reported were pain (53%, *n* = 112), restlessness/anxiety (62%, *n* = 132) and dyspnoea (47%, *n* = 99). A weak concordance was found regarding the documentation of symptoms between nurses and physicians (κ-coefficient 0.33 for pain, 0.21 for restlessness/anxiety and 0.50 for dyspnoea). This difference was observed in all departments (*p* < 0.01, respectively). Restlessness/anxiety were reported more often by nurses than physicians in all departments (*p* < 0.01, respectively). In terms of pain documentation, this difference was only noticed for non-oncological departments (*p* < 0.01).

In oncology, 90% (*n* = 72) of the pts with documented restlessness/anxiety received opioids. In all other departments, this was the case in 58% (*n* = 53) of pts (*p* < 0.01). Altogether, 88% (*n* = 112) of the pts with documented pain received an opioid. 13 of 15 pts who had not received an opioid despite documented pain were treated in non-oncology departments.

#### Multivariate analyses

The medical department was an independent factor for GPP* (*p* < 0.01). Pts dying in non-oncological departments had an increased risk not to get GPP* compared to pts in oncology: Pts dying in cardiology had a 4.2 times increased risk (95% CI 1.7–10.3; *p* < 0.01), in geriatrics a 5.3 times increased risk (95% CI 2.1–13.3; *p* < 0.01), in gastroenterology a 3.7 times increased risk (95% CI 1.7–8.2; *p* < 0.01) and in nephrology a 7.2 times increased risk (95% CI 2.4–21.6; *p* < 0.01).

Patients suffering from malignant diseases were 2.5 times more likely to receive GPP* than pts suffering from non-malignant diseases (95% CI 1.4–4.5; *p* < 0.01). Results of the logistic regression analysis are reported in Table 4.

**Table 4:** Multivariate analysis—“Patients who died on peripheral internal wards (*n* = 212) and good palliative practise*^a^”

Parameter	Estimate	Standard error	Wald Chi^2^	Pr > Chi^2^
Patients who received good palliative practise				
Malignant disease	0.9323	0.2908	10.2815	0.0013
Patients who received no good palliative practise:				
Medical department: cardiology	1.4363	0.4563	9.9102	0.0016
Medical department: geriatrics	1.6723	0.4663	12.8651	0.0003
Medical department: gastroenterology	1.3109	0.4030	10.5786	0.0011
Medical department: nephrology	1.9683	0.5634	12.2068	0.0005

^a^Both criteria had to be fulfilled for good palliative practice

^*^: use of opioids and the application of less than 1.5 L parenteral hydration

## Discussion

The current study was conducted to evaluate physician’s knowledge and attitudes regarding terminal care of dying pts. Moreover, we were interested in the analysis of pts records in terms of palliative terminal care to compare survey results and clinical data.

The response rate of 97% of physicians in our survey illustrates that the topic is highly relevant to physicians. A high amount of physicians polled in our survey felt uncertain and not adequately trained when caring for dying pts. In line with a systematic review about knowledge and management of pain in cancer pts by Makhlouf et al. in the year 2019, physicians with more experience in PC and oncology had fewer objections against opioids, which leads to the assumption that further improvement of teaching at medical schools, as well as improvement of PC trainings within fellowship, could be beneficial (Weber et al. 2016).

Our study results also revealed different knowledge of PC within the four medical departments as an influencing factor on the quality of terminal care. Multivariate analyses identified the medical department as a significant independent risk factor not to apply GPP/GPP* both, in the evaluation of the physician’s survey and the analysis of pts records. Physicians working in non-oncological departments were less likely to use GPP and the presence of malignant disease was an independent factor for GPP* in the analysis of the patient records. Recently published studies reported on shortcomings regarding knowledge of end-of-life symptom management in non-oncological specialties (Crousillat et al. 2018) which could lead to potential imbalances between pts in terms of end-of-life treatment outside of oncology (Clemans et al. 2014; Ernecoff et al. 2020). Moreover, PC is generally connected with the field of oncology what may lead to better awareness for and contact with PC among physicians working there. Although it is not surprising that physicians working in the oncology department were more familiar with the guideline for pts with incurable cancer, we would like to emphasize its importance for other departments as well. For example, 50% of pts treated in gastroenterology in this study had a cancer diagnosis.

As a consequence, terminally ill pts treated by physicians in the field of oncology might get higher amounts of PC measures than pts treated by physicians of other specialties. The observed difference in morphine dosage among all pts who received continuous intravenous administration in the last 48 h of life could be a hint for a connection between PC and oncology. The applied dose of morphine in oncology was lower than in the other internal departments but, in total, in oncology more pts received opioids and the therapy was started more frequently at earlier time points. This might be a consequence of a higher awareness leading to earlier treatment of symptom exacerbation—especially to symptoms occurring in the dying process. Nevertheless, some typical symptoms that might indicate the beginning of the dying process like restlessness/anxiety were more frequently documented by nurses than physicians in all departments. This emphasizes the need for concurrent inter-professional exchange between physicians and nurses leading to better multidisciplinary awareness of the medical condition of terminally ill pts.

Notwithstanding, the question of how to implement PC measures in daily work with terminally ill pts in all areas of medicine still remains unanswered. Various studies emphasize the need for integrating PC into clinical practice particularly with regard to non-oncological medical specialties (Verhofstede et al. 2017; Hua and Wunsch 2014; Gelfman et al. 2017). In the current study deviations from PC standards could be identified for terminally ill pts treated in all non-oncological internal departments, especially on ICUs. Even though nearly half of pts dying in ICU received best supportive care and no further life extending treatment, the majority of these pts received high amounts of artificial hydration or nutrition, which is not reflected by literature and the German S3 Guideline for Palliative Medicine (Hui et al. 2015; Leitlinienprogramm Onkologie 2019). To improve terminal care for dying pts in general, PC concepts should be integrated into treatment also on ICUs at earlier time points (Schuster et al. 2017). The German S3 Guideline for Palliative Medicine already provides recommendations for pts with incurable cancer (Leitlinienprogramm Onkologie 2019). Therefore, it should be rethought to develop recommendations for dying pts in general to ensure a high quality of care for all dying pts. Since typical symptoms as pain, dyspnea, and other symptoms also occur frequently in the dying phase of non-oncological pts and there is hardly any literature available, we partly assume that similar treatment is indicated. However, this also constitutes a limitation of our study and further research should be carried out regarding this topic as we cannot rule out deviations in the need for opioids between dying pts with or without malignant disease.

The data of the current investigation merely reflect one German university hospital. To be able to make more comprehensive statements, further studies including physicians, pts and additional medical professions are warranted. Prospective studies could increase the data quality, as the retrospective analysis of pts records in this study often revealed incomplete documentation of symptoms.

Different study settings including nursing homes and outpatient medical care might also improve quality of data and ease the further implementation of PC standards.

In conclusion, guideline-based PC for dying pts was found to be implemented more likely and consistently within oncology. There might be a natural bond between PC and oncology in general due to the high rate of none-curable disease causing higher awareness for PC measures and symptom management in terminally ill pts. We believe that PC should be integrated more consequently into medical training and everyday practice in other medical specialties to ensure good end-of-life care for all pts.

## Supplementary Information

Below is the link to the electronic supplementary material.

Supplementary file1 (PDF 190 KB)

## Data Availability

The authors confirm that the data supporting the findings of this study are available within the article and its supplementary materials.
