# Supplementary material for: Analysis of end-of-life treatment and physician perceptions at a university hospital in Germany
Source: J Cancer Res Clin Oncol. 2021 May 5;148(3):735–42. doi: 10.1007/s00432-021-03652-0 (PMC8881441; doi:10.1007/s00432-021-03652-0)
Supplement: Supplementary file 1 — Supplementary file1 (PDF 190 KB) [file 432_2021_3652_MOESM1_ESM.pdf]

Supplementary Information

Journal of Cancer Research and Clinical Oncology

Analysis of end-of-life treatment and physician perceptions at a university hospital in Germany

Nicole Heerde[1], Wolf Karsten Hofmann[1], Ralf Dieter Hofheinz[1], Sylvia Büttner[2], Deniz Gencer[1]

[1]3rd Department of Medicine, Medical Faculty Mannheim, University Medical Center Mannheim, Heidelberg University, Mannheim, Germany;

[2]Department of Medical Statistics, Biomathematics and Information Processing, University Medical Center Mannheim, Heidelberg University, Heidelberg, Germany

Corresponding author:

Nicole Heerde
III. Medizinische Klinik
Universitätsmedizin Mannheim
Universität Heidelberg
Theodor-Kutzer-Ufer 1–3
68167 Mannheim, Germany.
Tel: +49 621 383 4115
E-mail: ev227@stud.uni-heidelberg.de

Supplement 1:
The 21-item paper questionnaire:

a. Gender: ☐ male ☐ female

b. Medical specialty:
☐ 1st medical clinic: cardiology, angiology, pneumology
☐ 2nd medical clinic: gastroenterology, hepatology, infectiology, geriatrics
☐ 3rd medical clinic: hematology and internal oncology
☐ 5th medical clinic: nephrology, hypertensiology, endocrinology, diabetology, rheumatology

c. Position: ☐ fellow ☐ attending ☐ senior physician

d. Frequency of treating dying patients:
☐ daily
☐ at least once a week
☐ at least once a month
☐ less than once a month

1. In medical school, palliative medicine was sufficiently addressed.
☐ 1 – I fully disagree. ☐ 3 – I rather agree.
☐ 2 – I tend to disagree. ☐ 4 – I fully agree.

2. In my further education, palliative medicine was sufficiently addressed.
☐ 1 – I fully disagree. ☐ 3 – I rather agree.
☐ 2 – I tend to disagree. ☐ 4 – I fully agree.

3. I am familiar with the German S3 Guideline for Palliative Medicine.
☐ 1 – I fully disagree. ☐ 3 – I rather agree.
☐ 2 – I tend to disagree. ☐ 4 – I fully agree.

4. I comply with the WHO analgesic ladder when treating patients suffering from severe pain.
☐ 1 – I fully disagree. ☐ 3 – I rather agree.
☐ 2 – I tend to disagree. ☐ 4 – I fully agree.

5. I comply with the WHO recommendation for treatment of pain for cancer when treating patients with tumor pain, (“by mouth”, "by the clock", "by the ladder", "for the individual", "attention to detail").
☐ 1 – I fully disagree. ☐ 3 – I rather agree.
☐ 2 – I tend to disagree. ☐ 4 – I fully agree.

6. I usually feel certain and competent when treating dying patients.
☐ 1 – I fully disagree. ☐ 3 – I rather agree.
☐ 2 – I tend to disagree. ☐ 4 – I fully agree.

7. I often think about possible legal consequences discontinuing diagnostic and therapeutic measures in dying patients.
☐ 1 – I fully disagree. ☐ 3 – I rather agree.
☐ 2 – I tend to disagree. ☐ 4 – I fully agree.

8. Opioids should be used regularly to treat pain in dying patients.
☐ 1 – I fully disagree. ☐ 3 – I rather agree.
☐ 2 – I tend to disagree. ☐ 4 – I fully agree.

9. Opioids should be used regularly to treat other symptoms of dying patients, such as dyspnoea or anxiety.
☐ 1 – I fully disagree. ☐ 3 – I rather agree.
☐ 2 – I tend to disagree. ☐ 4 – I fully agree.

10. The side effects of opioids are usually negligible compared to the intended effect in treatment of dying patients.

☐ 1 – I fully disagree. ☐ 3 – I rather agree.
☐ 2 – I tend to disagree. ☐ 4 – I fully agree.

11. When treating severe pain or treating dying patients with opioids, the risk of developing tolerance and addiction is a problem.
☐ 1 – I fully disagree. ☐ 3 – I rather agree.
☐ 2 – I tend to disagree. ☐ 4 – I fully agree.

12. Opioids should always be used very carefully due to the risk of respiratory depression.
☐ 1 – I fully disagree. ☐ 3 – I rather agree.
☐ 2 – I tend to disagree. ☐ 4 – I fully agree.

13. There are major differences in the treatment of dying patients between malignant and non-malignant diseases.
☐ 1 – I fully disagree. ☐ 3 – I rather agree.
☐ 2 – I tend to disagree. ☐ 4 – I fully agree.

14. When treating dying patients, I often feel insecure whether the treatment is ideally for the patient.
☐ 1 – I fully disagree. ☐ 3 – I rather agree.
☐ 2 – I tend to disagree. ☐ 4 – I fully agree.

15. When treating dying patients, I also use other medications (except opioids) for palliative sedation (e.g. midazolam or other benzodiazepines).
☐ 1 – I fully disagree. ☐ 3 – I rather agree.
☐ 2 – I tend to disagree. ☐ 4 – I fully agree.

16. Dying patients should receive artificial hydration in order to avoid patients dying of thirst.
☐ 1 – I fully disagree. ☐ 3 – I rather agree.
☐ 2 – I tend to disagree. ☐ 4 – I fully agree.

17. Dying patients should receive artificial nutrition in order to avoid patients dying of starvation.
☐ 1 – I fully disagree. ☐ 3 – I rather agree.
☐ 2 – I tend to disagree. ☐ 4 – I fully agree.

Supplement 2: Patients who died on internal departments (n=278) - treatment within the last 48 hours of life

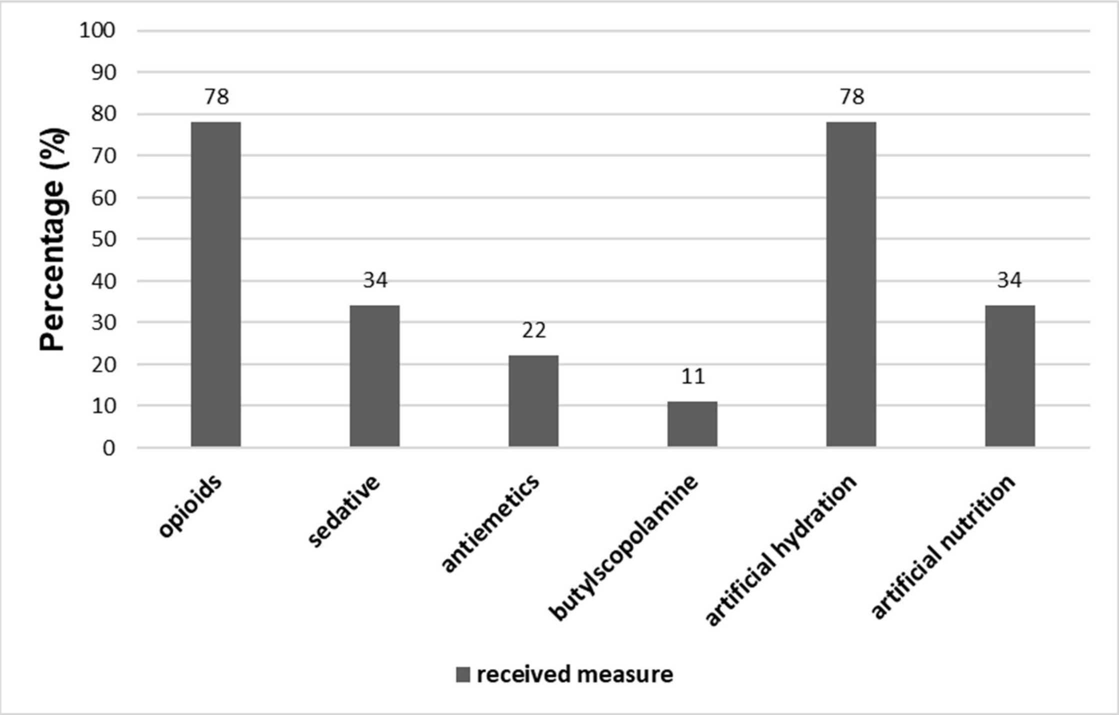

Supplement 3: Patients who died on internal departments (n=278) - diagnostic/invasive measures within the last 48 hours of life

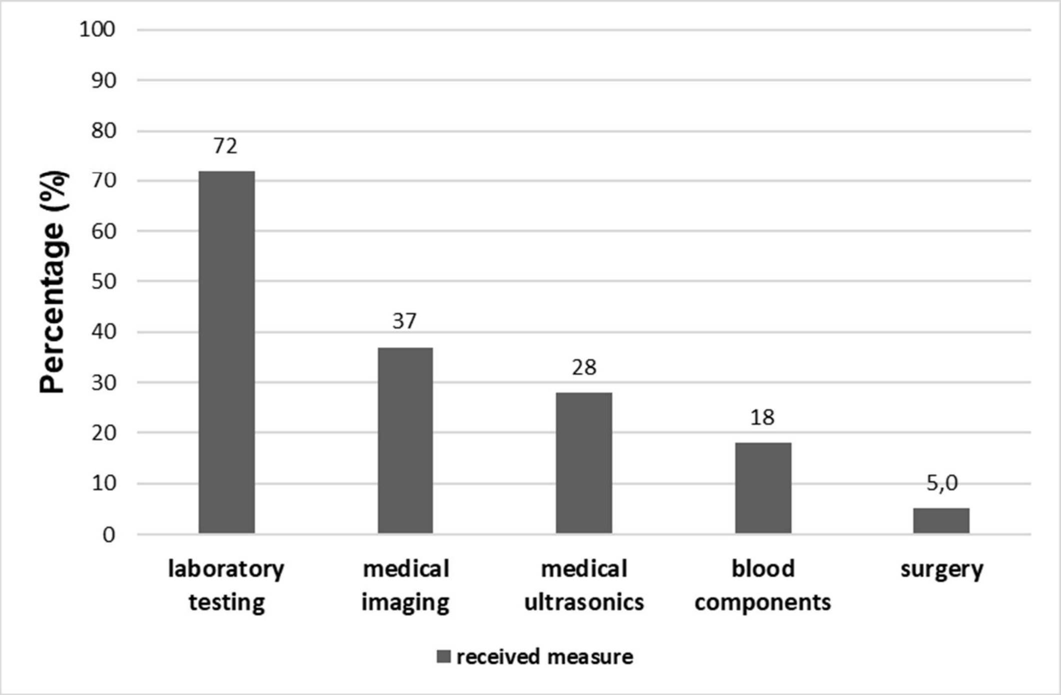

Supplement 4: Patients who died on peripheral internal wards (n=212) - documented symptoms in the last 48 hours of life

Symptoms	Documented at all n	%	Documented by physicians n	%	Documented by nurses n	%
Pain	112	53	68	32	88	42
Anxiety/restlessness	132	62	53	25	121	57
Dyspnea	99	47	66	31	84	40
Death rattle	38	18	17	8.0	32	15
Nausea	18	8.5	8	3.8	11	5.2
Escaping from bed	15	7.1	5	2.4	12	5.7
